# P-1198. Molecular and Microbiome/Metagenome Correlates of Illness in Respiratory Syncytial Virus (RSV) Infected Infants

**DOI:** 10.1093/ofid/ofae631.1381

**Published:** 2025-01-29

**Authors:** Mary T Caserta, Thomas Mariani, Steven R Gill, Ann Lindley Gill, Edward E Walsh, Anthony Corbett, Chin-Yi Chu, Xing Qiu

**Affiliations:** University of Rochester Medical Center, Rochester, New York; University of Rochester Medical Center, Rochester, New York; University of Rochester Medical Center, Rochester, New York; University of Rochester Medical Center, Rochester, New York; University of Rochester, Rochester, NY; University of Rochester Medical Center, Rochester, New York; University of Rochester Medical Center, Rochester, New York; University of Rochester Medical Center, Rochester, New York

## Abstract

**Background:**

Respiratory syncytial virus (RSV) epidemics are a leading cause of hospitalization in infants. Those infants with severe illness appear more likely to develop recurrent wheeze. We aimed to test if airway gene expression and microbiome/metagenome composition in the nasal epithelium during primary RSV infection are associated with illness severity and can identify infants with recurrent wheeze.

**Table 1.**
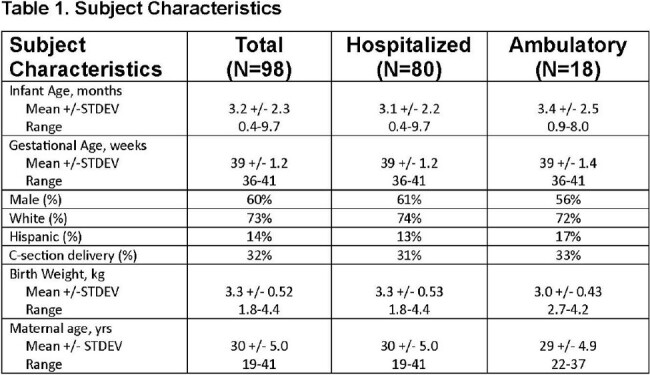
Subject Characteristics

**Methods:**

Healthy infants with PCR confirmed RSV were prospectively recruited from inpatient and outpatient locations over three seasons (Dec 2019 to Dec 2023). Clinical and demographic data, 2 anterior nasal swabs and a nasal wash were collected. Microbiome/metagenome analysis and transcriptome/RNA sequencing were performed on the nasal biospecimens. Disease severity was measured by the Global Respiratory Severity Score (GRSS) and retrained improved Global Respiratory Severity Score (iGRSS). Subjects were followed for 12 months to identify recurrent wheezing.

**Figure 1.**
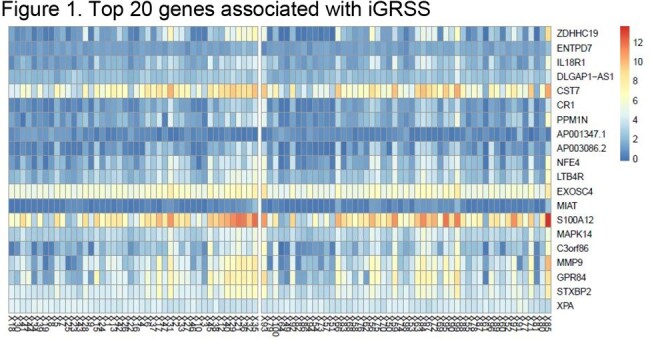
Top 20 genes associated with iGRSS

**Results:**

100 (80 hospitalized) infants were enrolled. Two children were found to be ineligible leaving 98 for analysis. The average age was 3.2 (2.3 SD) months. 60 (61%) were male. A multivariate linear regression model was used to associate gene expression profiles with GRSS and iGRSS. After controlling false discovery rate (FDR) at 0.10 level, 283 genes were significantly associated with iGRSS (95 at 0.05). Gene expression changes involved both Innate Immune and Interleukin Signaling pathways. Metagenome analysis identified microbial species and the microbial metabolic state of the nares during acute infection. We found an abundance of *Moraxella* in the anterior nares was inversely associated with iGRSS while the abundance of *Haemophilus* was directly associated with iGRSS. We also identified 29 metabolic pathways associated with the iGRSS including coenzyme A biosynthesis I (procaryotic), mixed acid fermentation, Cavin-Benson-Bassham cycle and superpathways of branched chain amino acid biosynthesis.

**Figure 2.**
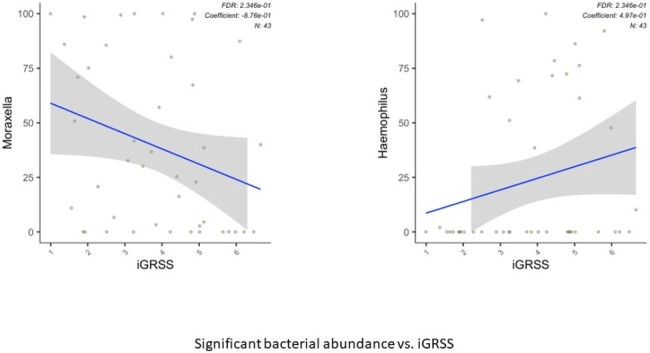
Bacterial Abundance vs. iGRSS

**Conclusion:**

We enrolled a cohort of previously healthy infants with primary RSV infection and identified associations between host nasal gene expression, nasal microbiome/metagenome composition/activity and disease severity. We are currently testing for associations between these biomarkers and recurrent wheeze.

**Figure 3.**
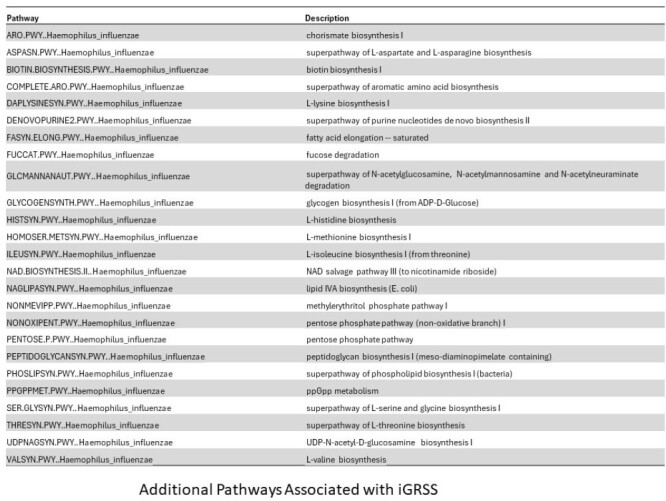
Metabolic Pathways associated with iGRSS

**Disclosures:**

**Mary T. Caserta, MD**, Merck: Grant/Research Support|Moderna: Grant/Research Support|Pfizer: Grant/Research Support **Edward E. Walsh, MD**, Enanta: Advisor/Consultant|Enanta: Honoraria|GSK: Advisor/Consultant|Janssen: Advisor/Consultant|Merck: Advisor/Consultant|Merck: Grant/Research Support|Pfizer: Advisor/Consultant|Pfizer: Grant/Research Support

